# An analysis of the influencing factors of depression in older adults under the home care model

**DOI:** 10.3389/fpubh.2023.1191266

**Published:** 2023-11-02

**Authors:** Yanjie You, Lei Huang, Xiao Peng, Lulu Liao, Fengjian Zhang, Mingjiao Feng, Yuqin Chen, Hongwei Chang, Beirong Mo, Yilan Liu

**Affiliations:** ^1^Department of Nursing, Union Hospital Affiliated to Tongji Medical College, Huazhong University of Science and Technology, Wuhan, China; ^2^School of Nursing, Tongji Medical College, Huazhong University of Science and Technology, Wuhan, China; ^3^Huazhong University of Science and Technology Union Shenzhen Hospital, Shenzhen, China

**Keywords:** depression, home-based care, self-care, population aging, mutual help

## Abstract

**Objectives:**

To explore and analyze the influencing factors of depression in older adults living at home, so as to propose suggestions for improving the quality of older adults living at home.

**Methods:**

We conducted a cross-sectional study on 498 older adults living at home based on questionnaire survey on the general information, daily living ability, health status, and care perception (including self-care, care for cohabitants, and care for non-cohabitants) of older adults living at home, as well as their willingness to help each other, and analyzed the influencing factors of depression among older adults living at home.

**Results:**

The results showed a willingness to help older adults, self-care, and total activities of daily living (ADL), health status was an influential factor for depression in older adults (*p* < 0.05).

**Conclusion:**

It aims to take targeted measures, such as encouraging older adults at home to actively participate in mutual assistance activities for older adults and care for themselves, so as to prevent and reduce the occurrence of depression in older adults.

## Introduction

Population aging has emerged as one of the major challenges facing the world. According to United Nations statistical data, the global population of people over the age of 65 is growing every year. Globally, the population is growing at a rate of 2% per year and is expected to account for about 16% of the global population aged over 65 in 2050, with a growing proportion of older people (1). In China, the increasingly severe and irreversible population aging is becoming the new national condition in the new era. According to the seventh population census in 2020, the population aged 60 and above has reached 260 million, with those aged 65 and above have reached 13% of the total population (2). Due to the dual role of productivity, economic and social development, and population birth control policy, China’s population transformation has been completed ahead of schedule, and population aging has developed rapidly, complicating China’s Decade of Action for Healthy Aging plan (3).

At present, depression has become a common mental disorder among older adults. Compared with young people, the course of senile depression is longer, and the treatment effect is worse (4). According to the World Health Organization (WHO) statistics, about 15–20% of adults over 60 years old have depression (5). Furthermore, depression will be the leading cause of the global disease burden by 2030 and one of the primary causes of disability and decline in quality of life in old age (6, 7). As a developing country, the health status of older adults in China is also not optimistic, with 33.1% of older adults suffering from depression (8). Even in some areas of China, the prevalence rate is higher than the statistics show (9). The high prevalence of depression among older adults may be due to their increased likelihood of social isolation and lack of communication, both of which contribute to negative emotions (10). At the same time, the weakening of physical function and the susceptibility to diseases can also lead to self-denial, pessimistic thoughts in older adults. Studies have shown that older adults with depression commit suicide, fast, refuse treatment, and other life-threatening behaviors (11, 12). It can be seen that senile depression will have great harm.

Despite the high incidence of depression among older adults, there are significant differences in the different forms or situations of older adults’ care. For example, Hua et al. (13) study showed that the prevalence of depression among older adults at nursing homes in China ranged from 13 to 30%, mainly because they felt lonely in a new environment far away from their families and homes. The incidence of depression among older adults in nursing homes is lower than the average, which could be because nursing personnel care for older adults in nursing homes, and institutions provide facilities and activities for them to enrich their recreational activities (14). According to the results obtained by Yin (15), approximately 22% of hospitalized older adults in China have symptoms of depression. The incidence of depression in the hospitalized older adults was lower than the average, which may be because they were cared for by professional medical staff, and their symptoms were relieved after treatment (16). According to the research (17) the leading causes of depression in hospitalized older individuals are new environmental diseases and economic pressure. However, older people living at home may be more prone to depression due to their lack of communication with the outside world. Simultaneously, unlike receiving careful care and social support during admission, older adults at home cannot receive professional care and help, are more likely to feel helpless, and are also likely to get depressed (18, 19). However, some researchers have opposite views, as some studies have shown that older adults at home can get more care from their children and maintain communication with neighbors (20). Moreover, the community provides volunteer service support to improve their quality of life, which can reduce the depression level of older adults at home (21). Thus, home care may not only have potential harm to the mental health of older adults but also may have beneficial promoting effects. Although some publications on depression status at home exist, they primarily focus on the depression status of older adults at home with chronic conditions and not fully comprehend the causes of emotional depression status at home. Therefore, it is necessary to timely understand the general depression tendency of older adults at home.

Furthermore, previous studies showed that many complex factors may be related to depression in older adults living at home, depending on the perspective of personal situations. For example, on a personal level, studies have shown that the incidence of depression in older women is higher than that in older men (22). Wu et al. (23) revealed that older adults living alone are likelier to feel lonely and cannot talk about negative emotions, so they are more prone to depression. Kugbey et al. pointed out that older adults are more likely to suffer from depression than the middle-aged and young older adults due to the impact of action and social detachment (8). However, other important demographic factors have not been mentioned, such as religious beliefs, number of children, and economic sources, etc. Regarding family factors, some studies have shown that caring for family members can reduce depression in older people. However, few researchers have shown whether the closeness and caring experiences of different family members potentially impact depression among older adults. Furthermore, the study also included an important variable, namely, activities of daily living (ADL), which can better reflect the most basic and common activities of older adults, including their ability to manage themselves. Tian et al. (21) research showed that basic activities of daily living (BADL) were associated with depression in older adults. However, few literatures have studied the effect of ADL on senile depression. At the social level, several studies have analyzed the influencing factors of depression in older adults from the perspective of social support. For example, due to the imperfect social support resources for older adults in China, they lack social support and are prone to feel lonely and social deprivation, leading to depression (18, 19). Although studies have shown that the spiritual comfort provided by community older adults’ care services can alleviate depression, few have conducted classified studies on the types of social support, such as community care institutions, volunteer associations, and other sources of care (20). Simultaneously, the older adults’ mutual aid service can improve depression, which has not been studied so far. However, few studies have explored the care of government departments, work units, friends, neighbors, medical institutions, and voluntary services, which can potentially alleviate depression in older adults at home. Therefore, the present study explores potential risk factors at various levels of individuals, families, and societies to provide a crucial foundation for developing more comprehensive intervention strategies.

In summary, this study aims to determine the prevalence of depression among home-dwelling older adults and the factors that may be associated with it. It will aid in developing multiple support strategies to reduce the risk of melancholy among older people living at home and to facilitate the adverse effects of their negative emotions on their mental health. Furthermore, the research results can provide a scientific basis for family carers, the government, and societies to employ effective intervention strategies.

## Materials and methods

### Object and method

#### Participants

The questionnaire of all participants is based on a survey of older adults at home in Hubei Province and Henan Province of China from March to May 2022, as well as the online questionnaire. All participants are surveyed.

Inclusion criteria: (1) Age ≥ 60 years; (2) The best way to care for older adults is to provide care at home; (3) There were no serious neurological and psychiatric diseases; (4) Could actively cooperate with the investigators and understand and express the contents of the questionnaire; and (5) Volunteered to participate in this study.

Exclusion criteria: (1) Older adults who did not agree to be investigated; (2) Older adults with communication difficulties; (3) Older adults who had mental retardation or severe mental disorder; (4) Older adults who had severe disabilities; and (5) Older adults who are approaching the end of their lives.

### Method

#### General information questionnaire

A self-designed questionnaire, which includes age, gender, educational background, whether living alone, number of children, number of chronic diseases, health status, living conditions, and other factors.

#### Patient health questionnaire-9 scale

PHQ-9 scale is one of the International General Depression Test Scales and the self-assessment Scale showed that can be used to screen people with depression. Williams and other researchers abroad believe that PHQ-9 has higher sensitivity and specificity than other depression screening tools. Furthermore, the PHQ-9 scale has no restrictions on age, gender, and race. In this scale, nine items covered the experience of happiness, depression, sleep interruption, energy level, appetite, feeling failures, inattention, slow speech or restlessness, and negative thinking about suicide or self-mutilation in the past 2 weeks (24). Items ranged from “0 = none” to “3 = almost every day”; A score of 0–4 indicates no depression, 5–9 indicates mild depression, 10–14 indicates moderate depression, 15–19 indicates moderate to severe depression, and 20–27 indicates severe depression.

#### Activities of daily living

Patients were assessed using the ADL scale and the Barthel index, with a score of 100 indicating that the patient can perform daily living activities well and does not need to rely on others. A score of 60 or more is considered good, indicating mild functional impairment; A score of 60 to 41 indicates a moderate functional impairment and the need for some assistance with daily living; A score of 40 to 21 indicates that the patient has a severe functional impairment; A score of less than 20 is considered fully disabled, indicating fully dependent on other people for daily living (25).

#### Home-based older adults’ perception of the caring survey

Self-design could explore the perception of humanistic care of the older adults. The scale used for this analysis has three dimensions, each with three items, and the three dimensions measure the self-care, cohabiting care, and non-cohabiting care of the older adults. The items are rated on a Likert-5 scale from “1 = never” to “5 = always.” A standardized formula was used to convert the total scale and individual dimension scores into a percentage score from 0 to 100, with higher scores indicating a stronger sense of care. Following the design, the questionnaire was finalized. After two rounds of expert consultation, revision, and reliability testing, Cronbach’s alpha coefficient was 0.945, with Cronbach’s alpha coefficient ranging from 0.811 to 0.941 for each dimension.

### Survey methodology

A cross-sectional survey was conducted, after obtaining the unification of older adults while accompanied by the investigator. A uniformly trained graduate student in nursing served as the investigator who conducted a one-on-one on-site survey of older adults who are living at home with the assistance of community neighborhood committee staff. Before the survey began, the staff of the neighborhood committee first led and explained the situation. During the survey, the investigator used unified instructional language, dictated the content of the questionnaire, and filled out the questionnaire face-to-face or online scanning of the older adults. To ensure an appropriate sample size for the multiple linear regression analysis, G power version 3.1.9.7 was used, assuming a significance level of 0.05, a median effect size of 0.15, a power power of 95%, and 12 predictors, the sample was calculated to be 184 participants. Allowing for a sample loss rate of 20%, at least 230 participants needed to be surveyed. A total of 516 questionnaires were eventually completed, with a effective response rate of 96.5% (26). The study was approved by the ethics committee of Huazhong University of Science and Technology (Ethical review No. S053), and all participants signed an informed consent form.

### Statistical methods

The SPSS 23.0 statistical software was used for the analysis. The measurement data conformed to the normal distribution described by (z ± s), and the count data were statistically characterized by frequency and composition ratio. Multiple linear regression was used to analyze the factors influencing the depressive mood of the older adults, with *p* < 0.05 considered a statistically significant difference.

## Results

### Basic information about older adults living at home

The 498 older adults in this study ranged in age from 60 to 98 years old, with an average age of (72.93 ± 8.39) years. The older adult participants in this study were 58.8% of females and 41.2% of males. Among these participants, who had the proportions with 0, 1, 2, and 3 or more children were 1.4, 25.9, 37.8, and 34.9%, respectively. Furthermore, 17.5% of the older adults were living alone, 74.9% had chronic diseases, 11.2% were in good health, 43.8% were in average health, and 45% were poor (see Table 1).

**Table 1:** Demographic characteristics, *N* = 498.

Age	72.93 ± 8.37
Sex-*N* (%)	
Female	293 (58.8)
Male	205 (41.2)
Number of children-*N* (%)	
0	7 (1.4)
1	129 (25.9)
2	188 (37.8)
≥3	174 (34.9)
Living alone, *N* (%)	
Yes	87 (17.3)
No	411 (82.3)
Chronic disease, *N* (%)	
Yes	373 (74.7)
No	125 (25.1)
Health condition, *N* (%)	
Good	224 (45.0)
Normal	218 (43.8)
Bad	56 (11.2)

### Occurrence of depression in older adults living at home

The older adults living at home had a PHQ-9 score of (16.58 ± 5.48). If the depression level was grouped based on the score, 31.1% had no depression, 41.6% had mild depression, 17.3% had moderate depression, 7.4% had moderate to severe depression, and 2.6% had severe depression. Among them, 30.1% of the total number of older adults aged 60–69 years old showed depression, 15.9% of those aged 70–79 years, and 22.9% of those aged greater than or equal to 80 years old (see Table 2).

**Table 2:** Occurrence of depression, *N* = 498.

Occurrence of depression		
Depression score	16.58 ± 5.48	
Degree of depression-*N* (%)		
No	155 (31.1)	
Mild	207 (41.6)	
Moderate	86 (17.3)	
Moderate to severe	37 (7.4)	
Severe	13 (2.6)	
Age-*N* (%)		
60–69 yrs	No depression	52 (10.4)
	Depression	150 (30.1)
70–79 yrs	No depression	56 (11.2)
	Depression	79 (15.9)
≥80 yrs	No depression	47 (9.4)
	Depression	114 (22.9)

### Univariate analysis of depression in older adults living at home

Following the chi-square test on the sample, the univariate analysis of depression in older adults with different characteristics was conducted. Here, the number of children (none = 0 1 = 1 2 = 2 3 = 3) willingness to help older adults (1 = unwilling 0 = willing) self-care, total ADL score, and health status (good fair poor) were related to older adults depression (Tables 3, 4).

**Table 3:** Univariate analysis of depression in older people with different characteristics at home.

Variable	*N* (%)	PHQ-9score	t/F	*p* value
Age			2.075	0.127
60–69 yrs	202 (40.6)	17.07 ± 5.357		
70–79 yrs	135 (34.1)	16.56 ± 5.842		
≥80 yrs	161 (25.3)	15.81 ± 5.12		
Sex			1.129	0.289
Female	293 (58.8)	16.27 ± 5.818		
Male	205 (41.2)	16.8 ± 5.233		
Marriage			0.141	0.935
Married	341 (68.5)	16.62 ± 5.549		
Unmarried	4 (0.8)	17.25 ± 12.685		
Divorced	5 (1.0)	15.2 ± 7.225		
Widowed	148 (29.7)	16.51 ± 5.06		
Education			2.554	0.079
Low	187 (37.5)	17.29 ± 5.675		
Moderate	240 (48.2)	16.15 ± 5.204		
High	71 (14.3)	16.15 ± 5.759		
Number of children			2.981	0.031
0	7 (1.4)	18.29 ± 9.604		
1	129 (25.9)	15.53 ± 5.127		
2	188 (37.8)	16.53 ± 5.461		
≥3	174 (34.9)	17.34 ± 5.471		
Number of diseases			1.689	0.168
0	125 (25.1)	15.72 ± 5.596		
1	216 (43.4)	16.83 ± 5.182		
2	94 (18.9)	16.57 ± 5.132		
≥3	63 (12.6)	17.43 ± 6.574		
Will to help the older adults			6.69	0.01
Will	361 (72.5)	16.97 ± 5.322		
Unwill	137 (27.5)	15.55 ± 5.779		
Health condition			12.701	0
Good	224 (45.0)	18.02 ± 6.454		
Normal	218 (43.8)	17.58 ± 5.332		
Bad	56 (11.2)	18.02 ± 6.454		
ADL score	96.93 ± 12.443	16.58 ± 5.482	1.793	0.029
Self-care	72.16 ± 14.766	16.58 ± 5.482	1.553	0.042
Cohabiting family care	45.78 ± 31.07	16.58 ± 5.482	1.473	0.07
Non cohabiting family care	74.17 ± 21.185	16.58 ± 5.482	1.533	0.55

**Table 4:** Multiple linear regression analysis.

Coefficient^a^								
Model		Unstandardized coefficient		Standardized coefficient	*t*	*p* value	Collinearity statistics	
		*B*	Standard error	Beta			Tolerance	VIF
1	Constant	25.186	1.894		13.301	0		
	ADL score	−0.089	0.019	−0.202	−4.582	0	1	1
2	Constant	28.563	2.065		13.834	0		
	ADL score	−0.077	0.019	−0.175	−3.978	0	0.975	1.026
	Self-care	−0.063	0.016	−0.169	−3.839	0	0.975	1.026
3	Constant	28.241	2.042		13.832	0		
	ADL score	−0.084	0.019	−0.192	−4.389	0	0.964	1.038
	Self-care	−0.068	0.016	−0.182	−4.174	0	0.968	1.033
	Will to help the older adults	1.924	0.531	0.157	3.62	0	0.979	1.022
4	Constant	27.123	2.059		13.171	0		
	ADL score	−0.086	0.019	−0.195	−4.512	0	0.963	1.039
	Self-care	−0.058	0.016	−0.156	−3.551	0	0.932	1.073
	Will to help the older adults	1.884	0.527	0.154	3.573	0	0.978	1.023
	Health condition-moderate	1.438	0.478	0.13	3.007	0.003	0.963	1.038
5	Constant	24.84	2.249		11.045	0		
	ADL score	−0.074	0.02	−0.168	−3.785	0	0.904	1.106
	Self-care	−0.051	0.017	−0.137	−3.085	0.002	0.904	1.106
	Will to help the older adults	2.139	0.535	0.174	4	0	0.941	1.063
	Health condition-moderate	1.888	0.51	0.171	3.703	0	0.839	1.191
	Health condition-bad	2.062	0.837	0.119	2.462	0.014	0.767	1.304

a Dependent variable: depression score.

### Multiple linear regression analysis of factors influencing depression in older adults living at home

The categorical variables were transformed into dummy variables according to needs, and the dummy variables are shown in the Table 5. Multiple linear regression was performed on the influencing factors that were statistically significant in the univariate analysis. This paper chose the stepwise method was chosen to conduct multiple linear regression of independent variables. The Pearson correlation test was conducted to examine the correlation between independent and independent variables for covariance. The results showed a willingness to help older adults, self-care, and total ADL. Health status was an influential factor for depression in older adults (*p* < 0.05). The number of children may have been excluded because of little correlation with other influencing factors.

**Table 5:** Assignment of the independent variable.

Will to help the older adults	Unwill = 0; Will = 1	
ADL score	Original value	
Self-care	Original value	
Number of children	0 = 0	
	1 = 1	
	2 = 2	
	≥3 = 3	
Dummy variable		
Health condition-good	0	0
Health condition-moderate	1	0
Health condition-bad	0	1

## Discussion

According to this study, 69.9% of older adults living at home had depressive symptoms. Some studies have shown that the rate of consultation among older adults experiencing depressive symptoms is much lower than the prevalence rate. This might be related to mental illness stigma, policy, culture, and self-neglect among older adults (27, 28). Older adults often choose to suffer alone because they believe it is a shame to know they have depression, lowering their quality of life in old age. In this study, age was found to have a folding relationship with the prevalence of depression. The 60- to 69-year-old older adults living at home had the highest incidence of depression since younger older adults have recently entered old age, their physical condition has deteriorated, their social role has changed from rich to single, they are not adapted to retirement life, and their social support has decreased (29, 30). The lowest incidence of depression among seniors aged 70 to 79 years may be related to role adaptation, acceptance of physical changes, and a more stable life situation; the rebound in the incidence of depression among seniors aged 80 years and above may be associated with increased loneliness and physical deterioration with increasing age (31, 32). Rich social activities should be organized for young and older adults to encourage participation in entertainment and continued contribution to society, thereby enriching their social responsibilities. For older adults, social volunteer organizations and family members should be organized to accompany and provide assistance in daily life, and nursing knowledge should be taught to slow down the deterioration of their physical condition.

Depression is associated with subjective health status in older residents. Researchers showed that factors related to subjective life perceptions rather than objective life conditions (i.e., gender, marital status, education, and chronic illness, etc.) might explain life satisfaction and depression in older adults (33). Furthermore, social support is positively correlated with the health status of older adults (34), implying that relevant communities, hospitals, and family members need to regularly monitor the health status of older adults to improve the quality of daily life at home and reduce their negative emotions caused by going out to see a doctor, taking medicine and inconvenient movement (35, 36). For older adults at home who have repeatedly visited the hospital and taken medicine for a long time, community hospitals should cooperate with the community to provide help and care for them at home regarding door-to-door visits, voluntary visits, and medication guidance. Government departments should provide home community hospital linkage services for older adults at home who have limited activities and lack the physical strength to provide a green channel for older adults to receive more convenient medical treatment and care.

The present study also found that the higher the self-care scores of homebound seniors, the lower the incidence of depression. Self-care in this study included “I will stay positive and optimistic,” “I will improve my health by regimen, such as exercising and eating healthy,” “I will comfort and enlighten myself especially when I am frustrated, and I will seek treatment and recovery especially when I need it,” “I will seek help from people around me especially when I am in trouble or feel helpless,” “I will dress up myself.” Self-care and self-care deficits exist to varying degrees among older adults, leading to a general lack of self-care skills among older adults (37, 38). Li et al. (39) showed that improving self-care effectively prevents depression in older adults. Since the degree of family care can affect the self-efficacy and self-management ability of older adults, family members of older adults at home should be called upon to motivate and help older adults to improve their self-care ability (40). Homebound seniors, especially those suffering from depression, are prone to self-neglect. Thus, it is clear that increasing self-care awareness among older adults may reduce depression. It is more important for the community and family members to remind the older adults to monitor their health and to provide timely care and assistance to those with low levels of self-care to improve their concerns for themselves.

The present study showed that higher scores of ADL were associated with a lower incidence of depression. The study by Liang and Penninxp found that ADL was a mediating effect of depressive symptoms in older adults and that depression was a risk factor for disability in the older population (41, 42). This may be because older adults living at home with high ADL scores can participate in more social activities, whereas older adults with low ADL scores have more difficulty taking care of themselves and require assistance from others, making them prone to feelings of frustration and helplessness (43). To prevent the deterioration of ADL in older adults with depressive symptoms, community care and social support should encourage them to exercise appropriately, eat a balanced diet, and maintain a positive attitude. For older adults with low ADL scores, psychological counseling and humanistic care should also be provided as early as possible, with regular psychological testing and observation of mood changes to prevent the occurrence of depression or reduce depressive symptoms and integrate them as much as possible into social activities.

Unlike other studies, this study showed that willingness to help older adults was associated with the level of depression and contributed the most. Older adults who were willing to help other older adults had a lower incidence of depression than those who were not ready to help others. Mutual aid for older adults has emerged as one of the new types of care occurring in various countries. In China, however, it is predominantly used to address the care for older adults’ requirements of empty nesters in rural areas, while its popularity in urban areas is low (44, 45). These studies showed that older adults who participate in volunteer activities could gradually identify their new roles, effectively improving their sense of well-being in life and thus reducing the occurrence of depressive symptoms (46). A study has suggested that mutual support among older adults may increase the opportunities for interpersonal interaction among older adults and may prevent the occurrence of depression (47). Simultaneously, studies have shown that older adults’ willingness to help each other may be related to their personality, social situation, and other factors (48, 49). People with cheerful and confident personalities communicate with others more frequently. Thus, they are more willing to help other older adults, whereas introverted older adults and social isolation tend to communicate less frequently with others and are prone to depression (50). As a result, encouraging mutual assistance in aging and allowing older adults living at home who are willing to age to participate in activities to help older adults will not only enable this older group to continue to play a role in society, participate in more social activities and gain social respect but will also provide early intervention or psychological guidance to those older adults who are in poor health and have a tendency to be depressed.

This study also has some limitations. The population in this study was mainly urban older adults living at home in Hubei and Henan provinces, which cannot represent the general older population. Since a proportion of older adults may have sleep disorders rather than depression-induced sleep disorders, the entries in the PHQ-9 scoring test may be misinterpreted.

In conclusion, geriatric depression was inextricably related to health status, self-care scores, willingness to help older adults, and ADL scores. In order to achieve healthy aging, which will enable homebound older adults to achieve a superior aging outcome, society and families should be mobilized to provide humanistic care support for older adults in all aspects of life, whether physiological or psychological. Consequently, older adults should be encouraged to participate in social activities as much as their bodies permit to get a warm lifestyle in their old age and reduce the incidence of depression.

## Limitation

We once added income in the pre experiment, but many older people are very sensitive to this and are reluctant to disclose their income for fear of being cheated. If the data is collected forcibly, it may significantly reduce the compliance of the older adults and then lead to the collection quality of other data lower than expected. For this reason, we deleted this item in the formal investigation stage and instead chose the payment method and income method to indirectly reflect this problem.

## Data availability statement

The raw data supporting the conclusions of this article will be made available by the authors, without undue reservation.

## Author contributions

YL, BM, YY, LH, and MF contributed to the conception of the study. YL, BM, YY, LH, YC, HC, and FZ performed the experiment. YY, FZ, XP, and LL contributed significantly to analysis and manuscript preparation. YY, LH, and LL performed the data analyses. YY and LH performed the drafting of manuscript. YL, BM, YC, HC, and XP revised the manuscript. LH, MF, YC, and HC performed the analysis with constructive discussions. All authors contributed to the article and approved the submitted version.
