# Supplementary material for: An analysis of the influencing factors of depression in older adults under the home care model
Source: Front Public Health. 2023 Nov 2;11:1191266. doi: 10.3389/fpubh.2023.1191266 (PMC10653336; doi:10.3389/fpubh.2023.1191266)
Supplement: Supplementary file 1 [file Data_Sheet_1.docx]

### ***General information questionnaire***

| What is your gender? | □Male □Femal | |
| --- | --- | --- |
| What is your age? | Years old | |
| What is your education level? | □No education □Primary school □Middle school □High school/Technical secondary school  □Junior college □Bachelor degree or above | |
| Do you have any religious beliefs? | □Yes □No | |
| What is your marital status? | □Unmarried □Married □Divorced □Widowed | |
| What was your main occupation? | □government-sponsored institution □Educators □medical personnel □Worker □Farmer □Soldier □Self-employed worker □Others | |
| What type of medical insurance do you have? | □Medical insurance for urban employees □Medical insurance for urban residents □New rural cooperative medical insurance  □Public medical care □Self-funded medical care □Others | |
| Where do you live? |  | |
| How many children do you have? | □None □1 □2 □≥3 | |
| Are your parents alive? | □Yes □One of them is alive □No | |
| Who do you live with? | □Alone □With wife or husband □With children □With support workers □With others | |
| Is there an elevator in your building? | □Yes □No（ Floor） | |
| How is your health? | □Very good □Good □Normal □Poor □Very poor | |
| By whom usually would you be cared for? | □Myself □Wife/Husband □Children □Support workers □Others | |
| Do you have chronic disease？ | □No □Yes(□Hypertension □Coronary disease □Stroke □Cancer □Diabetes □Chronic bronchitis □Asthma □Senile dementia □Arthritis □Others： | |
| Would you be willing to provide help and support to other older adults? | | □Yes □No |

### ***Patient Health Questionnaire-9 Scale (PHQ-9)***

| Are you not motivated or interested in doing things? | □Never □Few days a week □More than half a week □Almost everyday |
| --- | --- |
| Do you feel tired or not energetic? | □Never □Few days a week □More than half a week □Almost everyday |
| Do you have difficulty sleeping, restless or overslept? | □Never □Few days a week □More than half a week □Almost everyday |
| Do you have a poor appetite or eat too much? | □Never □Few days a week □More than half a week □Almost everyday |
| Do you have difficulty concentrating on things, such as reading newspapers or watching TV | □Never □Few days a week □More than half a week □Almost everyday |
| Do you move or speak slowly until others have noticed? Or on the contrary, become more fidgety or fidgety than usual? | □Never □Few days a week □More than half a week □Almost everyday |
| Do you feel bad or fail, or let yourself and your family down? | □Never □Few days a week □More than half a week □Almost everyday |
| Do you feel depressed or hopeless? | □Never □Few days a week □More than half a week □Almost everyday |
| Do you have the idea of not living or hurting yourself in some way? | □Never □Few days a week □More than half a week □Almost everyday |

### ***Activities of daily living (ADL)***

| When you walk on flat ground (within 45m)? | □Fully dependent on others □Need great help □Need some help □Independent |
| --- | --- |
| When you go up and down stairs? | □Fully dependent on others □Need great help □Need some help □Independent |
| When you take a bath? | □Fully dependent on others □Independent |
| When you go to the toilet? | □Fully dependent on others □Need some help □Independent |
| When you move in bed or wheelchair? | □Fully dependent on others □Need great help □Need some help □Independent |
| When you are dressing? | □Fully dependent on others □Independent |
| When you decorate? (such as washing) | □Fully dependent on others □Independent |
| When you are eating? | □Fully dependent on others □Need great help □Need some help □Independent |
| When you control your stool? | □Incontinent □Sometimes Incontinent □Normal |
| When you control your urine? | □Incontinent □Sometimes Incontinent □Normal |

### ***Home-based older adults’ perception of the caring survey***

| **A** Self-care |  |
| --- | --- |
| A1.1 Do you maintain a positive and optimistic attitude？ | □Never □Seldom □Sometimes □Often □Always |
| A1.2 Do you improve my health through health preservation, such as exercise and healthy diet？ | □Never □Seldom □Sometimes □Often □Always |
| A1.3 Do you comfort and enlighten myself, especially when I encounter setbacks and depression？ | □Never □Seldom □Sometimes □Often □Always |
| A1.4 Do you seek treatment and rehabilitation, especially when I am sick？ | □Never □Seldom □Sometimes □Often □Always |
| A1.5 Do you seek help from people around me, especially when I encounter difficulties or feel helpless？ | □Never □Seldom □Sometimes □Often □Always |
| A1.6 Do you cultivate or stick to my hobbies？ | □Never □Seldom □Sometimes □Often □Always |
| A1.7 Do you have a physical examination？ | □Never □Seldom □Sometimes □Often □Always |
| A1.8 Do you dress up myself？ | □Never □Seldom □Sometimes □Often □Always |
| **B** Caring from from cohabitation families |  |
| B1.1 Do cohabiting families talk to you and listen to you? | □Never □Seldom □Sometimes □Often □Always |
| B1.2 Do cohabiting families take care of your life? | □Never □Seldom □Sometimes □Often □Always |
| B1.3 Do cohabiting families accompany you to do things you want to do, such as entertainment, outing, dinner, etc? | □Never □Seldom □Sometimes □Often □Always |
| B1.4 Do cohabiting families care about your health, accompany you to see a doctor, and help you buy medicine? | □Never □Seldom □Sometimes □Often □Always |
| B1.5 Do cohabiting families comfort you and persuade you when you are distressed and sad? | □Never □Seldom □Sometimes □Often □Always |
| B1.6 Do cohabiting families celebrate with you on some important days, such as birthdays? | □Never □Seldom □Sometimes □Often □Always |
| B1.7 Do cohabiting families respect you? | □Never □Seldom □Sometimes □Often □Always |
| **C** Caring from non-cohabitating families |  |
| C1.1 Do non-cohabitating families care about you through phone or WeChat? | □Never □Seldom □Sometimes □Often □Always |
| C1.2 Do non-cohabitating families come to visit you and accompany you? | □Never □Seldom □Sometimes □Often □Always |
| C1.3 Do non-cohabitating families buy things for you, such as fruit, nutrition and daily necessities? | □Never □Seldom □Sometimes □Often □Always |
| C1.4 Do non-cohabitating families accompany you to do things you want to do, such as entertainment activities, outings, dinners, etc? | □Never □Seldom □Sometimes □Often □Always |
| C1.5 Do non-cohabitating families come to visit you and take care of you when you are sick? | □Never □Seldom □Sometimes □Often □Always |
| C1.6 Do non-cohabitating families settle down your work, family and life, and let you worry less? | □Never □Seldom □Sometimes □Often □Always |
| C1.7I Do you feel respected in the behavior of the younger generation? | □Never □Seldom □Sometimes □Often □Always |
